# Methylprednisolone stimulated gene expression (GILZ, MCL-1) and basal cortisol levels in multiple sclerosis patients in relapse are associated with clinical response

**DOI:** 10.1038/s41598-021-98868-y

**Published:** 2021-09-30

**Authors:** Maria Eleftheria Evangelopoulos, Narjes Nasiri-Ansari, Eva Kassi, Anna Papadopoulou, Dimitrios Stergios Evangelopoulos, Paraskevi Moutsatsou

**Affiliations:** 1 Department of Neurology, Eginition University Hospital, National and Kapodistrian University of Athens, Athens, Greece; 2 Department of Biological Chemistry, Medical School, National and Kapodistrian University of Athens, Athens, Greece; 3 Department of Clinical Biochemistry, School of Medicine, National and Kapodistrian University of Athens, University General Hospital Attikon, Rimini 1, Haidari, 12462 Athens, Greece

**Keywords:** Biochemistry, Molecular biology

## Abstract

Glucocorticoids (GCs) are the main treatment of relapse in multiple sclerosis (MS). Decreased sensitivity to GCs in MS patients has been associated with lack of the suppressive effect of GCs on inflammatory molecules as well as increased resistance to apoptosis. We investigated GC-sensitivity by measuring the effect of intravenous methylprednisolone (IVMP) treatment on transactivation of anti-inflammatory and apoptotic genes (GILZ, MCL-1 and NOXA respectively), in accordance to clinical outcome. Thirty nine MS patients were studied: 15 with clinically isolated syndrome (CIS), 12 with relapsing remitting (RRMS) and 12 with secondary progressive (SPMS) under relapse. Patients underwent treatment with IVMP for 5 days. Blood was drawn before IVMP treatment on day 1 and 1 h after IVMP treatment on days 1 and 5. GIlZ, MCL-1 and NOXA were determined by qPCR. The Expanded Disability Status was evaluated and patients were divided according to their clinical response to IVMP. GILZ and MCL-1 gene expression were significantly higher following first IVMP treatment in responders, compared to non-responders. Furthermore, serum basal cortisol and 1,25-OH Vitamin D levels were significantly higher in clinical-responders as compared to non-clinical responders. Our findings suggest that the differential GILZ and MCL-1 gene expression between clinical-responders and non-clinical responders may implicate the importance of GILZ and MCL-1 as possible markers for predicting glucocorticoid sensitivity and response to GC-therapy in MS patients following first IVMP injection.

## Introduction

Glucocorticoids (GCs) are widely used for the treatment of inflammatory autoimmune diseases, such as multiple sclerosis (MS). Glucocorticoids regulate MS at multiple levels: they suppress secretion of inflammatory mediators, such as cytokines, by T-lymphocytes and also eliminate the inflammatory T cells by inducing T-cell apoptosis. Intravenous high-dose exogenous GCs (methylprednisolone) are considered the standard therapy for acute relapse in MS patients^1^. However, patients with clinically isolated syndrome and relapsing MS demonstrate a better response to GC treatment compared to progressive MS (primary or secondary), suggesting inter-individual GC sensitivity differences in different MS sub-types^2^. Despite the importance of GCs in regulating key pathways of T lymphocytes (inflammation and apoptosis) in MS, the underlying mechanism(s) involved in treatment response are not known. More importantly, there are currently no prognostic markers to predict response to MP treatment.

GCs mediate their effects via their intracellular glucocorticoid receptor alpha and beta isoforms (GRα and GRβ respectively), both important regulators of HPA axis negative feedback and GC sensitivity. After binding to GCs, GRα dissociates from the heat shock proteins (HSPs), translocates into the nucleus where it homodimerizes and binds either to the positive or negative glucocorticoid response elements (GREs) in the promoters of target genes, resulting in upregulation or downregulation of gene transcription (direct transactivation or trans-repression respectively). Monomeric GRα may interact with other transcription factors such as CREB, AP-1, NF-kB or STAT, to repress or activate their target gene transcription (indirect trans-repression or transactivation). The GRβ does not bind to GCs and acts as a natural negative inhibitor of the GRα isoform^3^. Current evidence demonstrates that GCs mediate their anti-inflammatory and apoptotic effects in lymphocytes by indirect trans-repression (GRα/NF-kB pathway) as well as by direct transactivation (GRα/GRE) mode of action^4,5^.

Several lines of evidence support that MS patients exhibit hyperactive HPA axis, as assessed by the lack of response in the combined Dex-CRH suppression test, or by elevated basal or stimulated secretion of adrenocorticotropic hormone or cortisol^2,6,7^_,_ suggestive of GC resistance mainly at the progressive forms of MS^6,8^.

The presence of corticosteroid resistance in MS has also been demonstrated by evaluating the inhibitory effect of dexamethasone on cytokine production in stimulated peripheral mononuclear cells (PBMCs)^9,10^ as well as by the inhibitory effect of dexamethasone on T cell proliferation^11^. The association of GC resistance with the inflammatory disease activity^11^ indicates that modification of GC resistance might be a target for novel therapeutic strategies. Moreover, the assessment of the in vivo glucocorticoid sensitivity in MS using the expression of GR-target genes (GILZ, DUSP, FKBP) has revealed up-regulation of GR targeted genes in patients with mild to moderate disability, while patients with severe disability displayed gene transcription concentration lower than healthy controls^12^_,_ supporting further GC resistance in MS patients.

GILZ (Glucocorticoid—induced leucine zipper) is a GR transactivated target gene. It exhibits potent anti-inflammatory properties by suppressing pro-inflammatory cytokines and modulation of T cell action. In particular, GILZ is thought to regulate an immune response by modulating the Th1/Th2 balance and it has been suggested that could represent an immunomodulatory target in autoimmune diseases^13^. GILZ as well as other GR target genes such as the human Myeloid cell leukemia (MCL-1) and NOXA have crucial role in the regulation of GR-dependent apoptosis in lymphocytes^14–16^. Given the central role of GR signaling in mediating clinical response to GC therapy in MS (via regulation of inflammation and apoptosis), in the present study we measured key GR-target genes such as GILZ, MCL-1 and NOXA in MS patients during relapse and evaluated whether any of the abovementioned genes could be considered as markers of the MP treatment response. To further elucidate the mechanism responsible for the changes of these GR target genes and their role in clinical response, we also measured other parameters of GC signaling such as GR protein content and serum cortisol. Since recent data demonstrate that Vitamin D augments GC responsiveness in MS^17^, we also measured 1,25 OH Vit D levels .

## Material and methods

### Subjects

Thirty nine MS patients participated in this study. Fifteen (15) patients with clinically isolated syndrome (CIS), 12 patients with relapsing remitting MS (RR-MS) and 12 patients with secondary progressive MS (SPMS). All CIS patients and MS patients fulfilled McDonald criteria for clinically isolated syndrome (CIS) suggestive of MS and definite MS respectively^18^.

MS patients were recruited from 2008 to 2011 evenly throughout the years, from the MS Unit of the Neurology Department of Eginition University Hospital of Athens, as part of a larger study on serum neuroendocrine variables in MS. All patients gave informed consent for the participation in this study, approved by the Eginition Hospital Ethics Committee*.*

All patients were in acute relapse, hospitalized in the Day MS Clinic and were treated with Intravenous Methylprednisolone (IVMP) (1 g/day) for 5 consecutive days, followed by oral corticosteroid tapering. Relapse was defined as the occurrence of neurological symptoms with duration of at least 24 h, combined with an increase of at least 1.0 point on the Expanded Disability Status Scale (EDSS)^19^. MS patients were scored by the EDSS in relapse. before IVMP treatment and on the 5th day after treatment and 1 month after the initiation of IVMP therapy. All CIS patients were treatment-naive, while RRMS patients were on immunomodulatory treatment.

### Blood sample collection

Peripheral blood was drawn in the morning before IVMP treatment (Pre-MP) and 1 h after treatment (post-MP Day1) and also 1 h after IVMP the 5thday of their treatment (post-MP Day5).

Blood Serum samples were stored at − 70 °C until assayed.

### RNA extraction and quantitative

*GILZ, NOXA and MCL-1 mRNA levels were determined in MS patients before and after IVMP treatment* on day one (post-MP Day1) and day five (post-MP Day5) as follows.

Whole blood (500 μl) was directly transferred into RNA protect Animal Blood Tubes (76554, QIAGEN) and stored at − 80 °C. The mRNA extraction and cDNA synthesis were performed within 1 month after sample collection. Briefly, tubes were thawed and left for a further 2 h at room temperature to ensure efficient cell lysis. Total RNA extraction was performed using RNeasy Protect Animal Blood Kit (73224, QIAGEN), according to manufacturer’s instructions. After elution with 35 μL of RNase-free water, mRNA was stored at − 80 °C until further use. Concentration of all RNA samples was quantified by measuring the absorbance at 260 nm (A260) and 280 nm (A280) using Nanodrop ND-2000 spectrophotomer (Thermo Fisher Scientific, Waltham, MA). One (1) μg of total RNA was reverse transcribed using the iScript cDNA synthesis kit (170-8891, Bio-Rad), according to the kit manufacturer’s instructions. The cDNA was diluted with DNase & RNase free water in 1:5 ratio before applying to qPCR analysis. Quantitative real-time-PCR (qRT-PCR) was performed using CFX96 Touch Real-Time PCR Detection System (Bio-Rad). Ten (10) μl of iQ™ SYBR® Green Supermix (Bio-Rad), 4 μl of cDNA, and 300 nmol of primer set were used for amplification in a final volume of 20 μl. Expression of individual genes from each sample was normalized against the expression of housekeeping gene GAPDH. All real-time reactions were carried out in triplicate and the primers and the annealing temperature of each set used for amplification were as follows: GAPDH-Forward, 5′-GGGTGTGAACCATGAGAAGT-3′GAPDH-Reverse, 5′-CATGCCAGTGAGCTTCCCGTT-3′ (annealing 58 °C); GILZ-Forward, 5′-GGACTTCACGTTTCAGTGGACA-3′ GILZ-Reverse, 5′-AATGCGGCCACGGATG-3′ (annealing 58 °C); NOXA-Forward 5′-AAGAAGGCGCGCAAGAAC-3′ NOXA-Reverse 5′-TCCTGAGCA GAAGAGTTTGG-3′ (annealing 60 °C); MCL-1-Forward, 5′-TCAAAAACGAAGACGATGTGA-3′ and MCL-1-Reverse 5′-TCAAAAACGAAGACGATGTGA-3′ (annealing 58 °C). Each qPCR cycle included a denaturation step, 15 s at 95 °C; an annealing and extension step, 45 s at either 58 or 60 °C. A melting curve analysis was performed to confirm the specificity of quantitative polymerase chain reaction (qPCR) products.

Each qRT-PCR run included a negative control containing all reaction’s components but not cDNA template to detect contamination or non-specific amplification. The relative fold change was calculated according to the comparative CT (2^∆∆CT^) method^20^ after normalizing to the value before IVMP treatment (Pre-MP) for each patient.

### PBMCs isolation and protein extraction

Peripheral blood (16 ml) was collected in BD Vacutainer® CPT™ (362753, BD Biosciences) containing sodium heparin as an anticoagulant. Peripheral blood mononuclear cells (PBMCs) were isolated within 1 h after whole blood collection, according to the manufacturer’s instructions, by centrifugation at 1800 × G for 15 min, at room temperature within two hours of blood collection. After isolation, PBMCs’ pellet was re-suspended in BD pharma lysis buffer (555899, BD Biosciences) and incubated for 15 min at a dark chamber to lyse contaminating Red Blood Cells (RBCs) and then washed with PBS by centrifugation at 3000 rpm for 4 min. Cell viability in each sample was assessed by trypan blue exclusion. PBMC pellets were stored at − 80 °C until assayed. The PMBCs’ pellets were then lysed by adding the cell lysis buffer (9803, Cell Signaling) supplemented with PMSF prior to polyacrylamide gel electrophoresis (SDS-PAGE) and western blot analysis. Total protein concentrations were quantified by the Bradford dye-binding method^21^. Protein samples were stored at − 80 °C until time of assay.

### Western blot analysis

PBMCs’ protein extracts from MS patients were submitted to western blot analysis to determine whole cell GR protein levels. Western blot analysis was performed as previously described^22^. Briefly, thirty micrograms of proteins were loaded per lane and submitted to electrophoretic separation in a 10% SDS-PAGE gel and subsequently transferred to a nitrocellulose membrane (A5239, Applichem). The blotted membranes were blocked with PBS-T containing 5% skim milk for 1 h, at room temperature (RT). The blots were probed with appropriate concentration of primary antibodies against GR (3660, Cell Signaling, 1:1000) and β-actin (MAB1501, Millipore, 1:5000), overnight at 4 °C. The membranes were then washed with PBS-T and incubated for 1 h at room temperature in the presence of the appropriate horseradish peroxidase-conjugated secondary antibodies—Goat anti-Mouse IgG-HRP (31430, Thermo Scientific, 1:2500) and Goat anti-rabbit IgG-HRP (AP132P, Millipore, 1:2500). Detection of the immunoreactive bands was performed using enhanced chemiluminescence (ECL) system (170-5061, BioRad). β-actin served as a loading control. The intensity of the immunoreactive bands was quantified using Image-J densitometry software.

Protein extracts prepared from COS cells transiently transfected with GR-α plasmid were used as GR-α positive control. An aliquot of pooled standard sample was loaded in one lane of each gel. The pooled sample served as an internal standard to minimize the inter-assay variation for samples run in different gels^23^.

### Serum cortisol and total 1,25-hydroxyvitamin D assays

Determinations of Cortisol and total 1,25-hydroxy vitamin D levels were carried out in serum obtained from patients in the morning (8.00–8.30 am) of the first day before IVMP injection (Pre-MP). An Electrochemiluminescence Immunoassay (ECLIA-Roche Diagnostics) was used to measure serum cortisol and total vitamin D levels. Both Serum Cortisol and Total 1,25-hydroxyvitamin D were measured on the same day as blood collection.

Of note, all methods were carried out in accordance with relevant guidelines and regulations.

### Statistical analysis

Statistical analysis was performed with Prism 6.0 software (GraphPad). The nonparametric Mann–Whitney test or a 2-tailed paired t test was used for statistical comparisons. A value was considered significant with *P* < 0.05. All data are expressed as means ± standard deviations (SD).

### Ethics approval

This study was approved by the Eginition Hospital Ethics Committee*.*

### Consent to participate

All patients signed informed consent for the participation in this study.

## Results

### Subjects

Of the 39 patients participating in this study, 15 patients had CIS, 12 patients had RRMS and 12 patients had SPMS. All CIS patients were treatment naïve while 4/12 RRMS patients were on immunomodulatory treatment (3 received IFNb and 1 copolymer acetate) while 3/12 SPMS patients were treated with IFNb. MS patients were grouped into clinical responders (n = 25) and non-clinical responders (n = 14) according to their response to corticosteroids.

Demographic and clinical characteristics of the MS patients at baseline are presented in Table 1.

**Table 1:** Demographic and clinical characteristics of patients with MS.

MS subtype	Age mean ± SD	Sex Female/Male	Treatment	EDSS* mean ± SD
Clinically Isolated Syndrome (CIS) (n = 15)	34.10 ± 10	8/7	None	2.9 ± 0.77
Relapsing Remitting MS (RR-MS) (n = 12)	30.13 ± 6.6	7/5	3 patients under IFN-γ 1b and 1 patient under Copolymer acetate Treatment	2.6 ± 0.62
Secondary Progressive MS (SPMS) (n = 12)	50.29 ± 6.6	6/6	3 patients under IFN-γ 1b treatment	4.4 ± 0.44
Healthy Controls (n = 7)	37.57 ± 5.4	4/3	None	–

*EDSS expanded disability status scale.

### GILZ, MCL-1 and NOXA basal mRNA levels in different MS subtypes prior to MP treatment on Day 1

**Figure 1:**
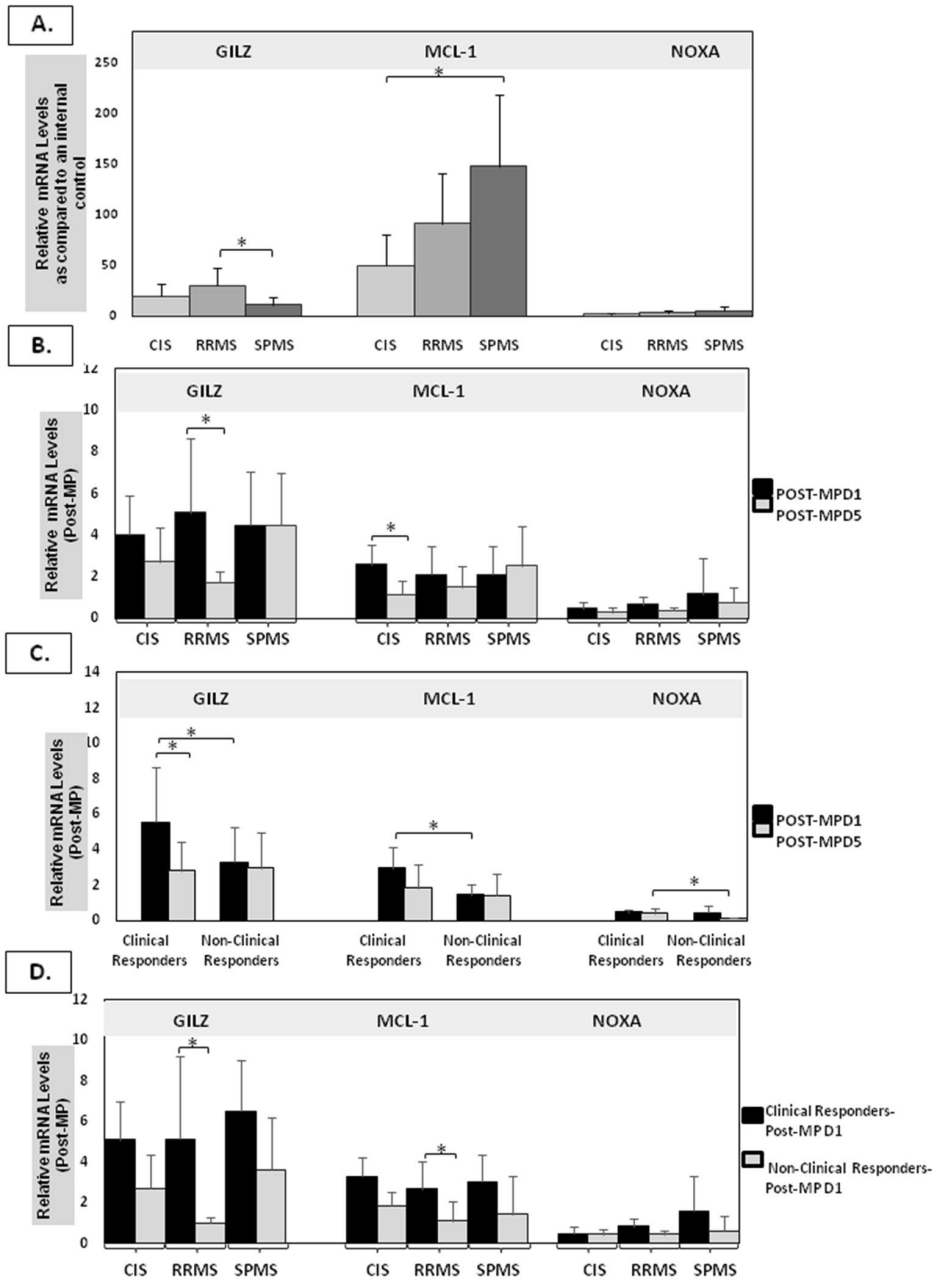
mRNA expression levels of GILZ, MCL-1 and NOXA genes. (**A**) Relative basal expression of GILZ, MCL-1 and NOXA in different MS subtypes before MP treatment on Day 1 acquired by RT‐qPCR. GILZ basal mRNA levels were significantly lower in SPMS (n = 12) as compared to RRMS (n = 12) group (*p* < 0.05) while MCL-1 mRNA levels was significantly induced in SPMS group as compared to CIS (n = 15) (*p* < 0.05). (n = sample size). (**B**) Relative expression of GILZ, MCL-1 and NOXA in different MS subtypes after MP treatment on Day 1 and Day 5 acquired by RT‐qPCR. GILZ and NOXA mRNA levels were significantly lower in RRMS (n = 12) after the 5th MP treatment as compared to values measured 1 h after the 1st MP treatment (*p* < 0.05). MCL-1 mRNA levels was also significantly reduced in CIS group 1 h after the 5th MP treatment as compared to values measured after the first MP treatment (*p* < 0.05). (**C**) GILZ and MCL-1 mRNA levels were significantly higher in clinical responders (n = 25) as compared to non- clinical responders (n = 14) after the 1st MP treatment. Moreover, the GILZ mRNA levels were significantly lower in clinical responders on Day 5 after 5th MP treatment as compared to values measured on Day 1. (n = sample size). (**D**) GILZ and MCL-1 mRNA levels were significantly higher in RRMS clinical responders (n = 9) one hour after the first MP injection as compared to non-clinical responders (n = 3) of the same group one hour after the 1st injection (*p* < 0.05). Data are shown as mean ± SD (*P < 0.05). (n = sample size).

For each patient, GILZ, MCL-1 and NOXA mRNA levels were measured before MP treatment (pre-MP), 1 h after IVMP—injection on the first day (PostMPD1) as well as 1 h after IVMP—injection on the fifth day (PostMPD5). The basal GILZ, MCL-1 and NOXA gene expression (mRNA levels) are expressed as fold increase, as compared to the expression of these genes in MCF-7 cell line. The basal GILZ, MCL-1 and NOXA mRNA levels in different MS subtypes are presented as mean ± SD in Fig. 1A.

Anova analysis showed a significant decrease in the basal *GILZ* mRNA levels in SPMS, as compared to RRMS group while the basal expression of *MCL-1* was elevated in SPMS, as compared to CIS patients (*p* < 0.05). No significant changes were observed in the basal mRNA levels of *NOXA* between CIS, RRMS and SPMS groups.

### GILZ, MCL-1 and NOXA mRNA levels in different MS subtypes after MP treatment on Day 1(PostMPD1) and Day 5(PostMPD5)

For each patient, *GILZ, MCL-1* and *NOXA*mRNA levels were measured before MP treatment (pre-MP), 1 h after IVMP injection on the first day (PostMPD1) as well as 1 h after MP IV injection on the fifth day (PostMPD5). Gene expression (mRNA levels) is expressed as fold increase, as compared to values measured (mRNA levels) before MP treatment (pre-MP). *GILZ, MCL-1* and *NOXA* mRNA levels on postMPD1 as well as on PostMPD5 for the different MS subtypes are presented as mean ± SD (Fig. 1B**).**

Anova analysis showed no significant difference in *GILZ, MCL-1* and *NOXA* mRNA levels between the CIS, RRMS and SPMS neither on PostMPD1, nor on PostMPD5 (*p* < 0.05 = ns).

For each subtype of MS patients, *GILZ, MCL-1* and *NOXA* mRNA levels on PostMPD1 were compared to those on PostMPD5.

For each patient, *GILZ, MCL-1* and *NOXA* mRNA levels were measured before MP treatment (pre-MP), 1 h after IVMP—injection on the first day (PostMPD1) as well as 1 h after IVMP injection on the fifth day (PostMPD5). Gene expression (mRNA levels) is expressed as fold increase, as compared to values measured (mRNA levels) before MP treatment (pre-MP). *GILZ, MCL-1* and *NOXA* mRNA levels on postMPD1 as well as on PostMPD5 for the different MS subtypes are presented as mean ± SD (Fig. 1B**)**.

Anova analysis showed no significant difference in *GILZ, MCL-1* and *NOXA* mRNA levels between the CIS, RRMS and SPMS neither on PostMPD1, nor on PostMPD5 (*p* < 0.05 = ns).

For each subtype of MS patients, *GILZ, MCL-1* and *NOXA* mRNA levels on PostMPD1 were compared to those on PostMPD5.

Paired t test analysis showed that *GILZ* mRNA levels were significantly lower on PostMPD5 as compared to PostMPD1 values only in RRMS patients, while there was no significant difference in CIS and SPMS patients (*p* = 0.04) (Fig. 1B).

Paired t-test analysis showed that *MCL-1*mRNA levels were significantly lower on PostMPD5 as compared to value measured on PostMPD1 in CIS group (*p* = 0.019) but not in RRMS or SPMS group.

### GILZ, MCL-1 and NOXA mRNA levels after MP treatment in clinical responders and non-clinical responders MS patients

During relapse, MP treatment may result in clinical improvement of MS patients. Therefore, we investigated whether *GILZ, MCL-1* and *NOXA* gene expression levels differ according to clinical response irrespective of the MS subtype.

qPCR analysis showed that the basal *GILZ* and *MCL-1* (Prior to IVMP on Day 1) mRNA levels were marginally higher in RRMS clinical responders as compared to RRMS non-clinical responders patients (*p* = 0.05 and *p* = 0.06 respectively) (Supplementary Fig. 1)*.* The basal *NOXA* mRNA levels didn’t differ between clinical responders and non-clinical responders of our study groups.

On PostMPD1, qPCR analysis showed that clinical responders had higher levels of *GILZ* and *MCL-1* mRNA levels, as compared to non-clinical responders (*p* = 0.02 and *p* = 0.004 respectively) (Fig. 1C)*. NOXA* mRNA levels didn’t differ between clinical responders and non-clinical responders on postMPD1.

On PostMPD5, no significant differences were observed in *GILZ, MCL-1*and *NOXA* mRNA levels, between clinical responders and non-clinical responders.

Paired t test analysis showed that the mRNA levels of *GILZ*in clinical responders was significantly lower on day 5, as compared to values measured on PostMPD1 (*p* = 0.019), but this was not the case for non-clinical responders.

No significant differences in *MCL-1* and *NOXA* mRNA levels were observed between PostMPD1 and PostMPD5, neither in clinical responders nor in non-clinical responders (Fig. 1C**).**

### GILZ, MCL-1 and NOXA mRNA levels after MP treatment in clinical responders and non-clinical responders within each MS subtype

We evaluated whether there was any difference in *GILZ, MCL-1*and *NOXA* mRNA levels after MP treatment depending on clinical response within each MS subgroup. On PostMPD1, RRMS clinical responders showed significantly higher *GILZ* mRNA levels, as compared to non-clinical responders (*p* = 0.03) (Fig. 1D, Table 1). However, *GILZ* mRNA expression in CIS and SPMS clinical responders did not differ significantly as compared to *GILZ* mRNA expression in non-clinical responders.

On PostMPD1, *MCL-1* mRNA levels were significantly higher in RRMS clinical responders compared to non-clinical responders (*p* = 0.02) (Fig. 1D). However, no significant differences were found in*MCL-1* mRNA expression levels between CIS and SPMS clinical responders and non-clinical responders (Fig. 1D, Table 1).

The *NOXA* mRNA levels were not significantly different between responders and non-responders in CIS, RRMS and SPMS patients on PostDay1 (Fig. 1D, Supplementary Table 1).

On PostMPD5, no significant difference was observed in *GILZ, MCL-1* and *NOXA* mRNA levels between clinical responders and non-responders, in the different MS subgroups. Additionally, we found no significant differences in *GILZ, MCL-1* and *NOXA* mRNA levels on PostMPD1 versus PostMPD5, neither in clinical responders nor in non-responders in CIS, RRMS and SPMS patients (Supplementary Table 1).

### Serum cortisol and 1,25 OH vitamin D levels

In order to test whether endogenous cortisol levels differ among MS patients before MP administration, we compared cortisol levels between different MS subgroups as well as cortisol levels between clinical responders and non-clinical responders.

**Figure 2:**
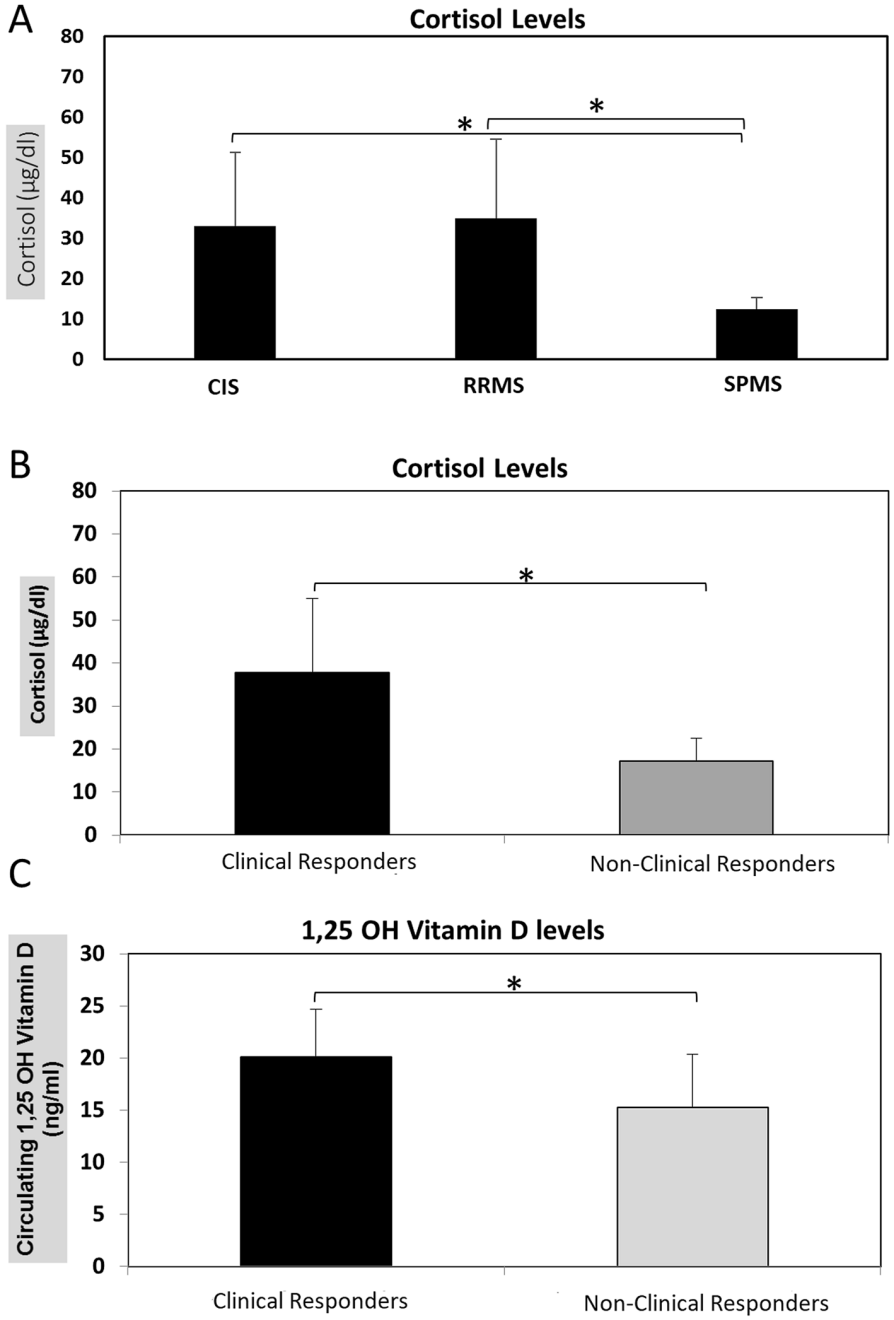
Serum Cortisol (μg/dl) and 1,25 OH Vitamin D levels (ng/ml) in MS patients 1 h before the 1st MP treatment. (**A**) Cortisol levels were significantly higher in CIS and RRMS as compared to SPMS patients (*p* < 0.05). (**B**) Cortisol levels is significantly higher in clinical responders as compared to non-clinical responders (*p* < 0.05). (**C**) The 1,25-Dihydroxyvitamin D levels are significantly higher in clinical responders as compared to non-clinical responders (*p* < 0.05).

All subjects had measurable baseline endogenous serum cortisol levels before IVMP administration on day 1. Serum cortisol concentration was significantly higher in CIS and RRMS compared to SPMS subtype (*p* = 0.04 and *p* = 0.04 respectively), whereas no significant difference was observed in endogenous serum cortisol level between CIS and RRMS subtypes (*p* = 0.6). (Fig. 2A).

Additionally, serum cortisol concentration in relapse was higher in clinical responders as compared to non-clinical responders before IVMP treatment, on day 1 (*p* = 0.03) (Fig. 2B).

Vitamin D3 levels did not differ significantly between MS subtypes (data not shown), whereas a marginal significant difference was observed between MS clinical responders and non-clinical responders (*p* = 0.05) (Fig. 2C).

### Glucocorticoid -receptor protein levels in MS patients

To evaluate the protein level of glucocorticoid receptor (GR) by western blot, 6 patients from each MS subgroup were randomly selected. A positive control lysate (COS cells transfected with GR-α) was used to demonstrate the specificity of the antibody for GR-α and for the recognition of the appropriate band size.

The CIS and RRMS patients (due also to their similar cortisol levels) were considered as one group and their GR levels were compared to SPMS patients’ group.

**Figure 3:**
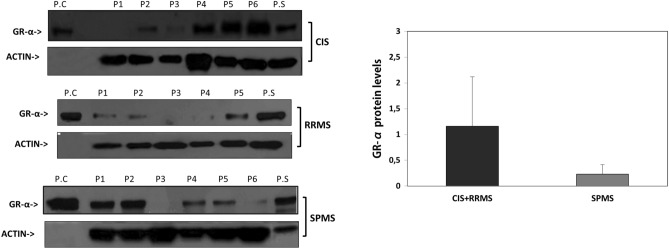
Glucocorticoid Receptor protein levels in MS patients. The GR-α Protein levels were marginally significantly higher in the group of CIS and RRMS patients compared to SPMS patients (*p* = 0.069). No GR-β protein was detected by Western blotting in our samples. No GR-β protein was detected by Western blotting in our experimental conditions. To evaluate the protein level of glucocorticoid receptor (GR) by western blot, 6 patients from each MS subgroups were randomly selected. A positive control lysate (COS cells transfected with GR-α) was used to demonstrate the specificity of the antibody for GR-α and for the recognition of the appropriate band size. Data are shown as mean ± SD (*P < 0.05).

The GR-α Protein levels showed a marginal significant difference between the group of CIS and RRMS patients with that of SPMS patients (*p* = 0.069). No GR-β protein was detected by Western blotting in our samples (Fig. 3).

## Discussion

Despite the wide use of corticosteroids (such as MP) to treat relapse in MS patients, the exact mechanisms via which GCs mediate their effects and regulate GC sensitivity and treatment response in MS are not fully elucidated. Moreover, there are currently no prognostic markers to predict response to MP treatment. Given the central role of GR in mediating clinical resistance and response to therapy, in this study we investigated whether GR-target genes could serve as markers of clinical response. *GILZ* and *MCL-1* are GR–target genes which are known to regulate inflammatory processes and apoptosis, both mechanisms involved in MS aetiopathogenesis and treatment^24^. In particular, GILZ is considered as a marker of GC sensitivity in vivo^25^, and it has been proposed that a GILZ based therapy could represent a way to control inflammation in SLE^26^.

The results of this study showed that MS patients in relapse, who clinically improved after iv methylprednisolone treatment (clinical responders), had significantly higher GILZ and MCL1 levels (more than twofold change) the first day of treatment. Interestingly, the expression of those two GR target genes was higher in RRMS patients who clinically improved with MP treatment. To our knowledge, this is the first study to report that clinical response following MP treatment is associated with higher GILZ and MCL-1 levels and that reduced induction of those two genes by MP is associated with no response to treatment. Additionally, our data support that GILZ and MCL-1 gene expression may be potential prognostic biomarkers of clinical response to MP treatment, in MS patients. Of note, SPMS patients had significantly lower basal GILZ and higher basal MCL-1 gene expression, as compared to RRMS and CIS respectively.

Importantly, basal GILZ and MCL-1 gene expression levels are higher (marginally) in RRMS patients who clinically improved after MP treatment, indicating that basal GILZ and MCL-1 values may be a potential prognostic biomarker of clinical response to MP treatment in RRMS patients.

Similarly, in SLE patients, a known systemic autoimmune disease, it has been shown that higher GILZ levels are associated with clinical response to glucocorticoid treatment, while reduced induction of GILZ by glucocorticoids is associated with increased disease activity, suggesting that GILZ induction is considered a marker of glucocorticoid treatment^13^.

In line with our investigation, De Anders et al., examined the expression of several genes in CD4 T lymphocytes of MS patients in relapse after MP treatment and found seven genes to be upregulated (DEFA4, CTSGI, DEFA8P, AZU1, MPO, ELANE, PRTN3) and four genes downregulated (IFNG, TNF, ZNF683) after IVMP administration^27^.

It is of interest that CTSGI, ZNF683 and MPO genes have glucocorticoid response elements in their promoter regions, thus explaining their differential expression after MP treatment. Pro-inflammatory genes such as TNF and IFNG were down regulated, as expected, due to the anti-inflammatory effect of MP. However, the above study did not examine the association of gene expression changes with clinical response, suggesting that future studies are needed to assess the role of these genes as MP treatment response biomarkers^27^.

Another study has examined the expression of GR target genes (GILZ, DUSP, FKBP5) in MS patients, in comparison to healthy individuals. They found GILZ to be significantly up-regulated in patients with mild or moderate disability compared to controls, while patients with severe disability showed GILZ concentrations to be lower than in healthy controls, suggesting the contribution of these genes in disease pathogenesis. They hypothesized that at the initial MS stages, HPA hyperactivity results in high cortisol levels, which in turn induce the expression of GR target genes, aiming to control neuroinflammation^12^.

In our study, we measured endogenous cortisol levels and found that were significantly increased in CIS and RRMS patients, types of disease characterized by neuroinflammation, compared to SPMS type which is characterized by lack of neuroinflammation and predominance of neurodegeneration. Our findings support that the increased cortisol response at the early disease stages could reflect a compensatory mechanism, aiming to control neuroinflammation, however this mechanism is down-regulated at the progressive stage of the disease.

In accordance to our findings, previous studies have shown that cortisol was significantly higher in RRMS in relapse, suggesting that elevated cortisol levels may reflect an endogenous response to neuroinflammation during relapse^28,29^.

Interestingly, our results revealed that increased cortisol levels characterized MS patients who clinically improved after MP treatment (clinical responders) compared to non-responders, indicating that in non-responders a dysregulation of the endocrine-immune communication is present. These data have not been previously reported and indicate that basal cortisol levels may be a prognostic factor to MP treatment response and disease progression.

Recent data have shown that 1,25 OH Vitamin D augments GC responsiveness in vitro and in vivo in MS^17^. Our data demonstrating that MS clinical responders have higher basal 1,25 OH Vitamin D alongside with higher basal cortisol levels (before MP treatment), support further the synergistic effects of Vitamin D and GCs to improve clinical response to GC treatment.

It is known that exposure to GCs affects differently transcription of responsive genes, an effect that depends on GC concentration and duration of exposure. For example, acute stress or short term exposure to GCs induces gene expression, whereas chronic stress and long exposure to exogenous glucocorticoids results in repression of GC-induced responsive genes (i.e. reduced gene induction), a phenomenon which depends on chromatin organization and GR binding to integrated targeted promoters^30^. In addition, chronic activation by GCs has been shown to lead to down regulation of glucocorticoids sensitivity in PBMCs^28^.

To this effect, we thought to compare the MP-induced GILZ expression on day 5 to that on day 1 in clinical responders and non-clinical responders. Paired t-test analysis revealed a significant repression of GILZ expression on day 5 compared to expression on day 1 in clinical responders, but not in non-clinical responders. Such data implicate a different chromatin organization and GR binding in target gene promoter between clinical responders and non-clinical responders group, which reflects the differential response to prolonged treatment (5 days). The repression of GILZ on the day 5 of MP treatment may be considered as a possible marker for predicting clinical response in further MP treatment.

Induction of T cell apoptosis in MS patients is considered to contribute to the therapeutic mechanisms of GCS^31^. In MS patients with non-clinical response, T cell apoptosis ex vivo has been shown to be lower than in clinical responders^17,32^.

In contrast to the above findings, in the current study, MP treatment resulted in upregulation of MCL1 gene, which is a known anti-apoptotic gene/protein. In particular MCL-1 gene was higher in clinical responders. The discrepancy between our results and those reported previously regarding apoptosis and clinical response in MS may be explained by recent findings supporting that survival of immune cells, both in vitro and in vivo, depends on the participation of multiple anti-apoptotic proteins rather than by a single anti-apoptotic protein^33^.

Our study has certain limitations. The size of our study group is limited. Therefore, our findings should be strengthened by future studies using larger number of treatment naïve patients. Although IFNb treatment has been shown to have no impact on the expression of GR downstream target genes (GILZ, DUSP, FKBP)^12^; an effect of IFN and copolymer acetate treatment on GILZ gene expression in RRMS and SPMS patients cannot be excluded.

In summary, the results of the current study provide first evidence that post MP values of GILZ and MCL-1 on the first day of treatment may be potential predictors of clinical response and effective MP treatment in MS. Basal cortisol and 1,25 OH Vit D serum levels may also serve to predict treatment response.

## Supplementary Information

Supplementary Figure 1.

Supplementary Table 1.

Supplementary Information.
