# Author Correction: Methylprednisolone stimulated gene expression (GILZ, MCL-1) and basal cortisol levels in multiple sclerosis patients in relapse are associated with clinical response

**DOI:** 10.1038/s41598-021-02415-8

**Published:** 2021-11-19

**Authors:** Maria Eleftheria Evangelopoulos, Narjes Nasiri‑Ansari, Eva Kassi, Anna Papadopoulou, Dimitrios Stergios Evangelopoulos, Paraskevi Moutsatsou

**Affiliations:** 1 Department of Neurology, Eginition University Hospital, National and Kapodistrian University of Athens, Athens, Greece; 2 Department of Biological Chemistry, Medical School, National and Kapodistrian University of Athens, Athens, Greece; 3 Department of Clinical Biochemistry, School of Medicine, National and Kapodistrian University of Athens, University General Hospital Attikon, Rimini 1, Haidari, 12462 Athens, Greece

Correction to: *Scientific Reports* 10.1038/s41598-021-98868-y, published online 30 September 2021

The original version of this Article contained an error in the order of the References 16 and 17, which was incorrectly given as:

16. Hoepner, R. *et al.* Vitamin D increases glucocorticoid efficacy via inhibition of mTORC1 in experimental models of multiple sclerosis. *Acta Neuropathol*. 10.1007/s00401-019-02018-8 (2019).

17. Ayroldi E, Riccardi C. Glucocorticoid‐induced leucine zipper (GILZ): a new important mediator of glucocorticoid action. *FASEB J* **23**, 3649–3658 (2009).

The correct order of the References is listed below:

16. Ayroldi E, Riccardi C. Glucocorticoid‐induced leucine zipper (GILZ): a new important mediator of glucocorticoid action. *FASEB J* **23**, 3649–3658 (2009).

17. Hoepner, R. *et al.* Vitamin D increases glucocorticoid efficacy via inhibition of mTORC1 in experimental models of multiple sclerosis. *Acta Neuropathol*. 10.1007/s00401-019-02018-8 (2019).

The original Article has been corrected.

