# Supplementary material for: Methylprednisolone stimulated gene expression (GILZ, MCL-1) and basal cortisol levels in multiple sclerosis patients in relapse are associated with clinical response
Source: Sci Rep. 2021 Sep 30;11:19462. doi: 10.1038/s41598-021-98868-y (PMC8484573; doi:10.1038/s41598-021-98868-y)
Supplement: Supplementary file 1 — Supplementary Figure 1. [file 41598_2021_98868_MOESM1_ESM.docx]

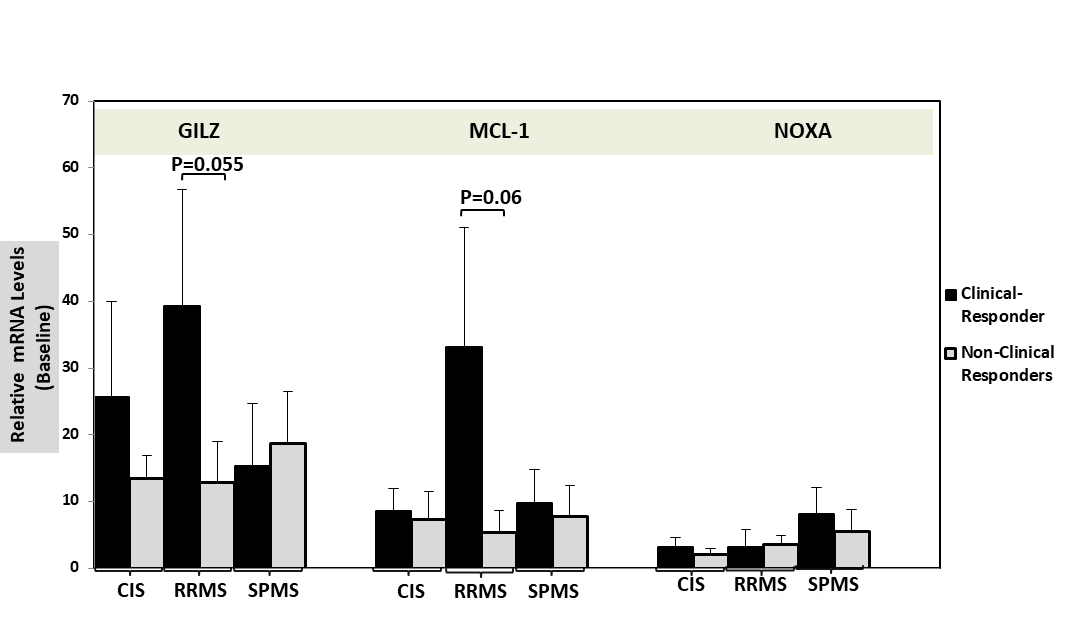


**Supplementary Figure 1. Basal mRNA levels of GILZ, MCL-1 and NOXA genes in clinical-responders and non-clinical responders of each MS patients sub groups.**

GILZ and MCL-1 mRNA levels were marginally higher in RRMS clinical responders (n=9) as compared to RRMS non-clinical responders (n=3) prior to the 1st MP treatment. (n=sample size).
