# Supplementary material for: Methylprednisolone stimulated gene expression (GILZ, MCL-1) and basal cortisol levels in multiple sclerosis patients in relapse are associated with clinical response
Source: Sci Rep. 2021 Sep 30;11:19462. doi: 10.1038/s41598-021-98868-y (PMC8484573; doi:10.1038/s41598-021-98868-y)
Supplement: Supplementary file 2 — Supplementary Table 1. [file 41598_2021_98868_MOESM2_ESM.pdf]

“Methylprednisolone stimulated gene expression (GILZ, MCL-1) and basal cortisol levels in multiple sclerosis patients in relapse: association with clinical response”

Maria Eleftheria Evangelopoulos*
Department of Neurology, Eginition University Hospital, National and Kapodistrian University of Athens, Athens, Greece.
Email: evangelopoulos@yahoo.com

Narjes Nasiri-Ansari*
Department of Biological Chemistry, Medical School, National and Kapodistrian University of Athens, Greece.
Email: nnasiri@med.uoa.gr

Eva Kassi
Department of Biological Chemistry, Medical School, National and Kapodistrian University of Athens, Greece.
Email: evakassis@gmail.com

Anna Papadopoulou
Department of Clinical Biochemistry, National and Kapodistrian University of Athens, School of Medicine,
University General Hospital Attikon, Rimini 1, Haidari, 12462, Athens, Greece. Email: Anpapado@med.uoa.gr

Dimitrios Stergios Evangelopoulos
Department of Biological Chemistry, Medical School, National and Kapodistrian University of Athens, Greece.
Email: ds.evangelopoulos@gmail.com

Paraskevi Moutsatsou
Department of Clinical Biochemistry, National and Kapodistrian University of Athens, School of Medicine,
University General Hospital Attikon, Rimini 1, Haidari, 12462, Athens, Greece.
Department of Biological Chemistry, Medical School, National and Kapodistrian University of Athens, Greece.
Email: pmoutsatsou@med.uoa.gr

*** Maria Eleftheria Evangelopoulos and Narjes Nasiri-Ansari are joint first authors.**

Corresponding author:

Paraskevi Moutsatsou
Department of Clinical Biochemistry, National and Kapodistrian University of Athens, School of Medicine,
University General Hospital Attikon, Rimini 1, Haidari, 12462, Athens, Greece.
Department of Biological Chemistry, Medical School, National and Kapodistrian University of Athens, Greece.
Email: pmoutsatsou@med.uoa.gr,
Tel/fax : 0030 -10-5832359/ 10-5831912

Supplementary Table 1. The expression of GILZ, MCL-1 and NOXA in each MS subtype, grouped according to their clinical response to IVMP therapy.

MS-subtype	Blood Sampling after MP administration	Clinical-Responders mRNA levels (mean ±SD)	Non-Clinical Responders mRNA levels (mean ±SD)	P- Value
**GILZ**				
CIS	POST MP-DAY 1	5.1±1.95	2.7±0.27	0.11=ns
	POST MP-DAY 5	2.6±0.89	2.34±2.51	0.28=ns
RRMS	POST MP-DAY 1	5.16±4.56	1.01±0.02	0.035=s*
	POST MP-DAY 5	1.78±0.23	1.9±0.36	0.392=ns
SPMS	POST MP-DAY1	6.52±2.95	3.65±2.17	0.22=ns
	POST MP-DAY5	6.5±0.7	2.75±2.21	0.4=ns
**MCL-1**				
CIS	POST MP-DAY1	3.29±0.852	1.86±0.35	0.028=s*
	POST MP-DAY5	1.32±0.61	0.94±0.77	0.2=ns
RRMS	POST MP-DAY1	2.69±1.43	1.09±0.33	0.14=ns
	POST MP-DAY5	1.57±1.21	1.09±0.33	1=ns
SPMS	POST MP-DAY1	3.04±1.54	1.42±0.79	0.22=ns
	POST MP-DAY5	2.94±1.91	2.13±2.14	1=ns
**NOXA**				
CIS	POST MP-DAY1	0.49±0.13	0.44±0.42	0.15
	POST MP-DAY5	0.43±0.27	0.14±0.03	0.28
RRMS	POST MP-DAY1	0.83±0.28	0.45±0.46	0.53
	POST MP-DAY5	0.39±0.135	0.33±0.19	0.71
SPMS	POST MP-DAY1	1.61±2.42	0.61±0.45	0.9
	POST MP-DAY5	0.86±0.93	0.59±0.37	0.9
