# Supplementary figures and images for: Methylprednisolone stimulated gene expression (GILZ, MCL-1) and basal cortisol levels in multiple sclerosis patients in relapse are associated with clinical response
Source: Sci Rep. 2021 Sep 30;11:19462. doi: 10.1038/s41598-021-98868-y (PMC8484573; doi:10.1038/s41598-021-98868-y)

CIS

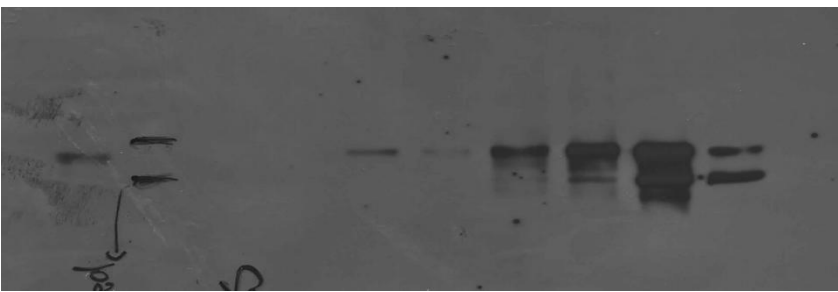

GR-α

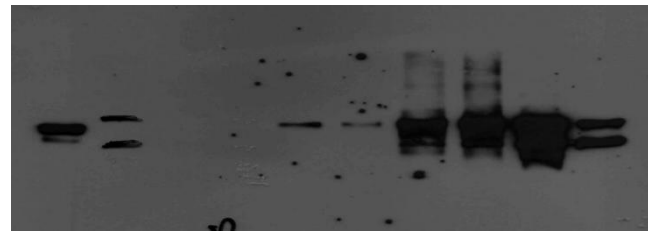

Longer exposure time

ACTIN

RRMS

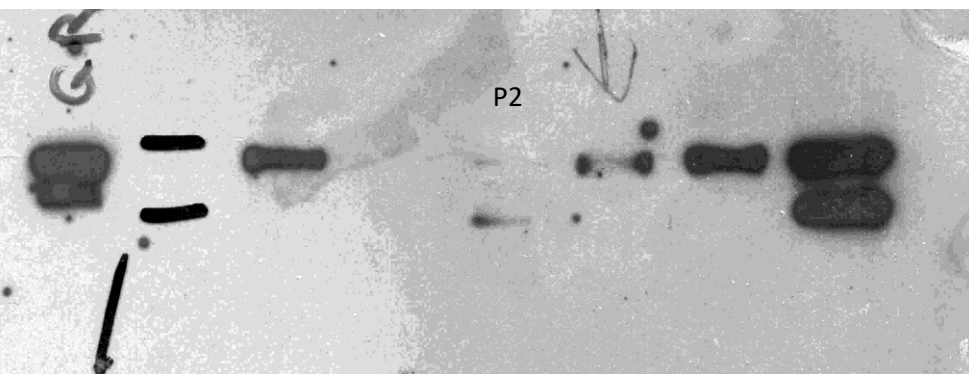

GR-α

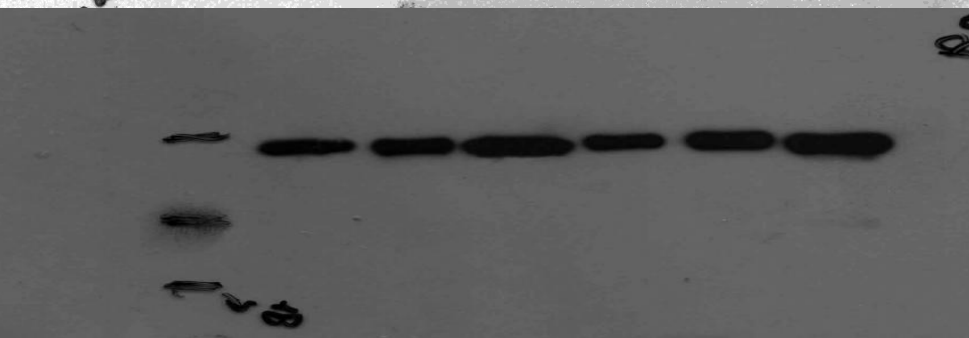

ACTIN

SPMS

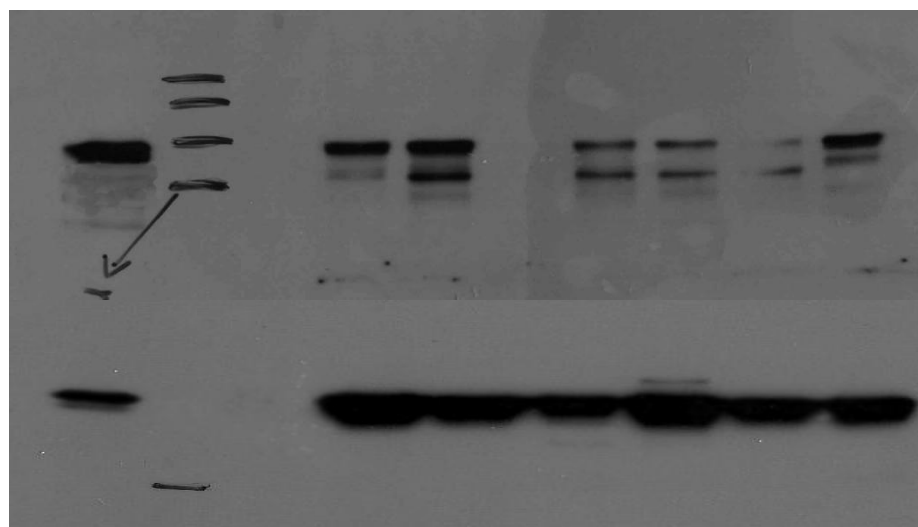

GR-α

ACTIN

Supplement: Supplementary file 3 — Supplementary Information. [file 41598_2021_98868_MOESM3_ESM.pdf]
